# Effect of high-fidelity simulation on alpha-amylase activity and concentrations of secretory immunoglobulin class A, cortisol, and testosterone among medical students

**DOI:** 10.1007/s12020-021-02696-z

**Published:** 2021-04-05

**Authors:** Szymon Bialka, Maja Copik, Adam Ubych, Radosław Marciniak, Jacek Smereka, Lukasz Szarpak, Hanna Misiolek

**Affiliations:** 1 Department of Anaesthesiology, Intensive Care and Emergency Medicine, Faculty of Medical Sciences in Zabrze, Medical University of Silesia, Katowice, Poland; 2 Center of Didactics and Medical Simulation, Medical University of Silesia, Katowice, Poland; 3 Laboratory for Experimental Medicine and Innovative Technologies, Department of Emergency Medical Service, Wroclaw Medical University, Wroclaw, Poland; 4 Bialystok Cancer Center, Bialystok, Poland

**Keywords:** High-fidelity simulation, Physiological stress, Stress hormones, Stress, Medical education

## Abstract

**Purpose:**

High-fidelity simulation calls heavily upon cognitive capacities and generates stress and anxiety. The objective of this prospective, observational study was to evaluate the degree of stress in medical students by measuring hormone levels during critical care classes.

**Methods:**

Overall, 55 students (senior years of medical faculty) of both sexes were divided into 5-person teams. Demographic data and information on diagnosed diseases, stimulants used, and previous experience in the field of medical simulation were collected with a personal questionnaire. Before starting the scenario (T0), after the end of the scenario (T1), and 120 min thereafter (T2), stress level was measured. For this purpose, systolic blood pressure, diastolic blood pressure, mean blood pressure, heart rate and blood oxygen saturation were evaluated. In addition, saliva was collected to determine alpha-amylase activity and the concentrations of secretory immunoglobulin class A, cortisol, and testosterone.

**Results:**

Among hemodynamic parameters, systolic and mean blood pressure and heart rate were significantly higher in T1 than in T0 and T2 time points (*p* < 0.05). Cortisol concentration was higher at T2 compared with T0 and T1. Alpha-amylase activity was highest at T1. Secretory immunoglobulin class A concentration was highest at T0, followed by T1 and then T2. These differences were not statistically significant. Testosterone concentration showed significantly higher values at T2 compared with T0 and T1 (*p* < 0.05). The analysis of team leaders vs. other members revealed significantly lower cortisol and alpha-amylase values in leaders (*p* < 0.05).

**Conclusions:**

High-fidelity simulation is a useful education method in medical subjects, especially in cases where a mistake could produce serious or irreversible consequences. It can increase stress hormone concentrations and thus can be assumed effective as a learning aid even in senior-year students of medical faculty.

## Introduction

High-fidelity simulation (HFS) fills the gap between standard, theoretical classes and practical training in clinical subjects, especially in specialties that need increased caution like anesthesiology [1]. This is why HFS is widely applied in teaching anesthesia, intensive care, and emergency medicine. There is evidence in literature that proves it an effective aid in gaining knowledge and skills. Moreover, HFS can induce behavior that can be beneficial for real patients [2]. A substantial advantage of HFS as an educational tool is that learners can practice on multiple levels (cognitive, procedural, and affective) in a protected environment, where the risk of error will not harm real patients [3–6].

Medical simulation induces stress, both psychological and physiological. It seems that the stress is even more pronounced than that associated with traditional teaching or even daily clinical work [7, 8].

Established methods of evaluating stress include measurement of heart rate (HR) and blood pressure (BP) [9]. Biochemical markers of stress are also known. Most of them are associated with the autonomic nervous or immune systems. Among the markers, there are alpha-amylase, cortisol, and testosterone. Alpha-amylase is a major salivary enzyme in humans, secreted in response to sympathetic stimuli [10]. Its activity increases under psychological stressors [11]. Cortisol is a stress hormone produced in the adrenal cortex, and its concentration in saliva is correlated with its concentration in blood plasma [12]. Other hormonal changes due to exposure to stress include lowering testosterone concentration [13]. Next to cortisol, alpha-amylase, and testosterone, also secretory immunoglobulin A (sIgA) was reported to be a stress marker that reflects mental stress. A study revealed that sIgA concentration continuously decreased in the presence of stress and that the sIgA reduction persisted even after the removal of stress factors [14].

We aimed to evaluate if HFS induced stress in students of 5th and 6th year of medicine faculty and what the stress level was. Salivary alpha-amylase activity and concentrations of cortisol, testosterone, and sIgA were used as stress markers. In addition, we measured HR, BP, and blood oxygen saturation (SpO_2_) of the participants.

## Material and methods

### Subjects

Overall, 55 medical faculty students (5th and 6th year; 28 women, 27 men) scheduled to participate in HFS as part of a standard scholastic program were enrolled in the study. Participation was voluntary. Written informed consent was obtained prior to inclusion. The inclusion criterion was the willingness to take part in the study. Exclusion criteria involved known pregnancy, active infections, immune system diseases, metabolic or endocrine disorders, and the current use of any medication (except for oral contraceptives).

### Study design

The study, designed as a prospective, observational trial, was conducted at the Center of Didactics and Medical Simulation of the Medical University of Silesia (Katowice, Poland) between April and June 2017. It was approved by the Ethics Committee of the Medical University of Silesia (approval No.: KNW/0022/KB1/35/1/17) and registered retrospectively at ClinicalTrials.gov (registration No.: NCT04381572).

At the beginning of the scheduled classes in the simulation center, in the morning (9:00–12:00), the students (5 per simulation) were asked to sit at rest for 30 min. After this time, their basic stress levels (T0) were established. In each team, a leader was chosen (having the most experience in the field of medical simulation). Other team members were also assigned detailed functions (2 assistants and 2 nurses). At the same time, data on sex, age, weight, height, diagnosed diseases, stimulants used, and previous experience in the field of medical simulation were collected with a personal questionnaire. We also made sure that conditions of the experiment were comparable—students were asked to sleep min. 8 h the previous night, have a light meal and avoid other stressors.

Before starting the simulation scenario, the participants were oriented for 10–15 min by a physician instructor about the simulation room setup and manikin features. Immediately after the end of the scenario (T1), the stress level was measured again. Then, the students were asked to sit at rest for 120 min and the stress level was determined once more (T2).

The simulation scenarios were implemented with the use of high-fidelity computer-based manikin simulators allowing for a remote control of vital signs (SimMan 3G, SimBaby, and SimJunior; Laerdal, Norway). All medications and equipment required during the clinical training were available. Standardized physiological responses to the anticipated management steps were programmed and activated by a physician instructor. When an unexpected clinical decision was taken by a participant, the physiological response was entered manually by the monitoring physician. The applied scenario was prepared and validated by experienced simulation instructors.

### Scenario design

A 40-year-old man was transported to the emergency department. He was confused, with suspected carbon monoxide poisoning that had occurred at home (the whole family suffered). During the scenario, the patient’s condition worsened: he developed heart arrhythmias and had fluctuating levels of consciousness. After 5 min, the next paramedical team brought in a 4-year-old boy (HR: 70 bpm; BP: 65/20 mm Hg; broad, stiff pupils; and no response to peripheral pain stimulation). One minute after admission, the child underwent a cardiac arrest (asystole). At that time, the adult patient had become nervous and aggressive. After another 5 min, the third paramedical team arrived with an 11-month-old infant with cardiac arrest. The man was becoming even more nervous and aggressive. He developed critical hypertension (SBP 210 mmHg, DBP 125 mmHg, MBP 153 mmHg, HR 130 bpm, SpO2 82%) with subsequent cardiac arrest (ventricular fibrillation). Until the end of the scenario, the resuscitation of the adult man and the 4-year- and 11-month-old children continued. Total duration of the scenario was 30 min.

### Data collection and analysis

At each of the 3 time points (T0, T1, and T2), HR [bpm], systolic blood pressure (SBP) [mm Hg], diastolic blood pressure (DBP) [mm Hg], mean blood pressure (MBP) [mm Hg], and oxygen saturation (SpO_2)_) [%] were assessed with the use of a cardiac monitor (Infinity Delta; Dräger, Germany).

At the same time, saliva was collected for immunoassay tests, performed with the Salivette system (Sarstedt AG & Co., Germany). A sterile tampon was positioned under the tongue or chewed for 30–45 s. The saliva-soaked pad was then transferred into a suspended insert with a perforated bottom. The insert with the tampon was placed in a centrifuge tube and closed with a stopper. Next, the tube was centrifuged (1000 × *g* for 10 min) to obtain a ready-to-test saliva supernatant. Approximately 0.7 ml of the supernatant from each collected sample was used for further testing. After centrifugation, the samples were frozen at –85 °C until performing laboratory tests. The saliva supernatant was tested for alpha-amylase activity, and the concentrations of sIgA, cortisol, and testosterone.

The alpha-amylase activity assay was performed with a static method by using an Amylaza kit (Aqua-Med, Poland). The samples were diluted 100 times with 0.9% chloride solution. 2-chloro-4-nitrophenyl-maltotrioside is a substrate in this method. The reaction was performed in pH 6.0 MES buffer at 37 °C and rendered a colored reaction product, which was then analyzed via spectrophotometry at 405 nm. The results are presented in salivary alpha-amylase activity units [U/ml]. The measurement imprecision of the method is 4.1%.

The concentration of sIgA was evaluated with an enzyme-linked immunosorbent assay (ELISA) (Immundiagnostik AG, Germany). The analytical procedure followed the manufacturer’s instructions presented in the user manual attached to the kit. The absorbance readings were taken by using a µQuant reader (BioTek, USA); the results were processed with the KC Junior software (BioTek, USA). The method is characterized by 2.5 µg/ml sensitivity and 5.3% imprecision.

A commercial ELISA kit (Diapra, Italy) was used to determine the concentration of cortisol and testosterone. The analytical procedure was in accordance with the manufacturer’s instructions provided in the manuals supplied with the kits. The absorbance readings were taken with a µQuant reader (BioTek, USA); the KC Junior software (BioTek, USA) served to process the results. The sensitivity of the method is 0.12 ng/ml for cortisol and 3.28 pg/ml for testosterone. The imprecision equals 6.2% and 7.9%, respectively.

### Statistical analysis

Data with normal distribution are presented as mean ± standard deviation (SD). Qualitative data are presented as n (%). Normal distribution of the presented data was evaluated by the Shapiro–Wilk test. For comparison between the groups, student *t* test was used for independent variables (homogeneity of variances was tested with Levene’s test) and Mann–Whitney U test for other data. A recursive weighted least squares estimation method was used for fitting a regression model of variability of studied data overtime. A *P* value < 0.05 was considered statistically significant. *P* values were corrected with Bonferroni correction for multiple comparisons. Data were analyzed with Statistica 13.0EN and MS Office Excel.

## Results

### Demographic characteristics

The study involved 55 students of both sexes. Their demographic characteristics are presented in Table 1.

**Table 1:** Characteristics of the participants

Parameter	Mean
Male sex [*n* (%)]	27 (48.2%)
Age [years]	25 ± 2.7
Body mass index [kg/m^2^]	22.4 ± 3.0
Previous simulations [*n* (%)]	20 (36.4%)
Completed Basic Life Support course [*n* (%)]	31 (66.1%)
Good assessment of own preparation [*n* (%)]	11 (19.6%)

Data presented as mean ± SD or *n* (%)

### Stress response

Contrary to what was expected, the difference in variables between the groups and measured in predefined time points was not very pronounced.

Among hemodynamic parameters, SBP, MBP, and HR were higher at the T1 than at the T0 and T2 time points (*p* < 0.05). The statistical threshold for significance was achieved, however taking under consideration the size effect, the difference was small as in all measurements the numbers did not reach above values considered as normal.

**Fig. 1:**
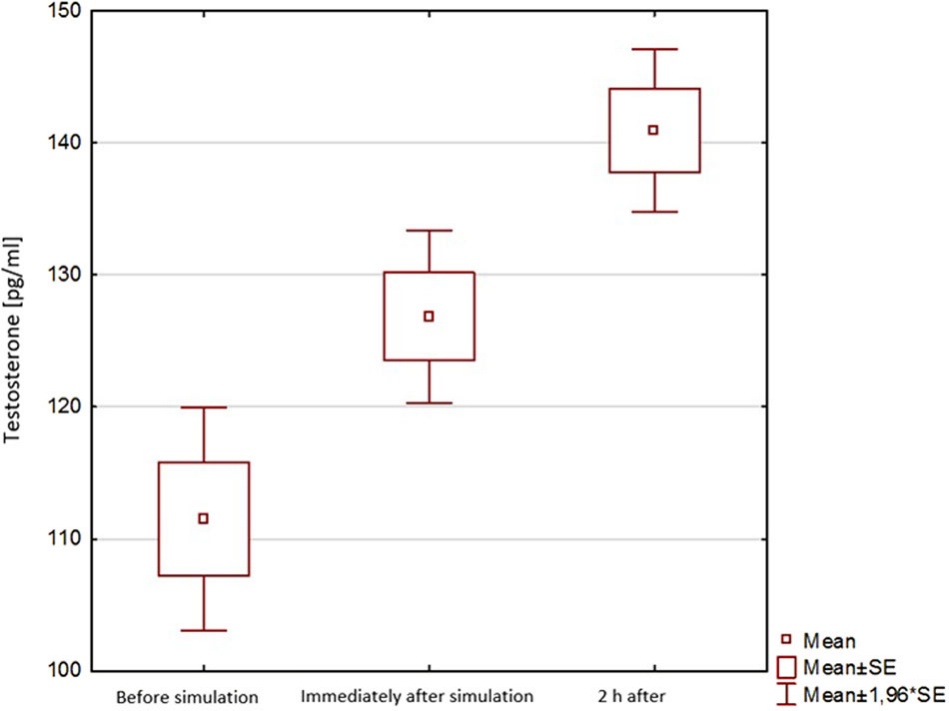
Univariate comparison of testosterone concentration before and after simulation

Regarding stress hormone levels the situation was quite similar. We recorded some differences in measured parameters, specifically cortisol concentration was higher at T2 compared with T0 and T1 and Alpha-amylase activity was highest at T1—immediately after the simulation. The concentration of sIgA was highest at T0, followed by T1 and then T2, however these differences were not statistically significant and one cannot confidently state that such small difference can indicate a reaction to a stressful stimuli (Table 2). The only variable that showed a significant difference was testosterone concentration which was higher at T2 compared with T0 and T1 (*p* < 0.05) (Fig. 1) but after careful observation it is obvious that the concentration did neither vary between the sexes, nor compared between team leaders and the rest of the group.

**Table 2:** Univariate comparison of clinical data before and after simulation

Parameter	T0	T1	T2	*p*
SBP [mm Hg]	126 ± 13	132 ± 11	123 ± 11	<0.001
DBP [mm Hg]	73 ± 10	76 ± 10	72 ± 10	0.067
MBP [mm Hg]	94 ± 10	98 ± 9	92 ± 9	<0.001
HR [bpm]	81 ± 13	89 ± 17	76 ± 12	<0.001
SpO_2_ [%]	98 ± 1	98 ± 1	98 ± 1	0.071
Cortisol [nmol/l]	102.1 ± 33.3	104.6 ± 28.2	106.8 ± 31.5	0.726
Testosterone [pg/ml]	111.5 ± 32.2	126.8 ± 24.9	140.9 ± 23.6	<0.001
Alpha-amylase [U/ml]	51.6 ± 14.9	56.3 ± 14.9	51.8 ± 15.6	0.186
sIgA [µg/ml]	229.4 ± 47.7	227.2 ± 50.3	220.1 ± 46.9	0.573

Data presented as mean ± SD. T0 – time point before starting the scenario, T1 – time point after the end of the scenario, T2 – time point 120 min thereafter

*SBP* systolic blood pressure, *DBP* diastolic blood pressure, *MBP* mean blood pressure, *HR* heart rate, *SpO2* blood oxygen saturation, *sIgA* secretory immunoglobulin A, *TP* total protein

Table 3 presents a comparison of the evaluated parameters depending on sex.

**Table 3:** Characteristics of the study group by gender

Parameter	Females		Males		*p*
	Mean	SD	Mean	SD	
Age [years]	24.8	1.2	25.2	3.7	0.557
Previous simulations [*n*]	22.1	17.4	17.5	14.3	0.324
BMI [kg/m^2^]	22.2	2.8	22.6	3.3	0.593
T0 point					
SBP [mm Hg]	122	12	131	13	0.007
DBP [mm Hg]	74	9	73	11	0.706
MBP [mm Hg]	93	9	95	10	0.460
HR [bpm]	81	14	80	12	0.722
SpO_2_ [%]	99	1	98	1	0.001
Cortisol [nmol/l]	102.2	35.1	102.0	31.8	0.985
Testosterone [pg/ml]	115.0	29.7	107.7	34.9	0.404
Alpha-amylase [U/ml]	52.0	14.4	51.3	15.6	0.851
sIgA [µg/ml]	228.8	49.7	230.0	46.5	0.923
T1 point					
SBP [mm Hg]	128	12	136	10	0.010
DBP [mm Hg]	76	9	75	11	0.792
MBP [mm Hg]	98	9	99	9	0.671
HR [bpm]	89	16	89	19	0.920
SpO_2_ [%]	98	1	97	1	0.007
Cortisol [nmol/l]	107.2	28.5	101.8	28.3	0.483
Testosterone [pg/ml]	132.2	22.9	121.0	26.1	0.093
Alpha-amylase [U/ml]	55.2	13.1	57.5	16.9	0.565
sIgA [µg/ml]	228.1	49.1	226.1	52.6	0.882
T2 point					
SBP [mm Hg]	117	10	128	10	0.000
DBP [mm Hg]	72	9	71	10	0.672
MBP [mm Hg]	90	8	94	9	0.120
HR [bpm]	77	14	76	11	0.923
SpO_2_ [%]	99	1	98	1	0.005
Cortisol [nmol/l]	107.6	30.8	106.0	32.8	0.847
Testosterone [pg/ml]	135.0	23.9	147.3	21.8	0.050
Alpha-amylase [U/ml]	51.5	16.0	52.1	15.4	0.890
sIgA [µg/ml]	217.8	44.8	222.7	49.6	0.697

Data presented as mean ± SD. T0 – time point before starting the scenario, T1 – time point after the end of the scenario, T2 – time point 120 min thereafter

*BMI* body mass index, *SBP* systolic blood pressure, *DBP* diastolic blood pressure, *MBP* mean blood pressure, *HR* heart rate, *SpO2* blood oxygen saturation, *sIgA* secretory immunoglobulin A, *TP* total protein

Raw data suggests that males had statistically significantly higher SBP and lower SpO_2_ values at each time point, but as above the size of the effect is small and its clinical significance is negligible. We did not find any difference in measured laboratory parameters that would be clinically significant.

We also did an additional analysis comparing haemodynamic and laboratory stress reaction parameters in team leaders vs. other members (Table 4 - Supplementary Material) that revealed significantly lower cortisol and alpha-amylase concentrations in leaders (*p* < 0.05), while the concentration of sIgA was higher among other team members (*p* < 0.05) but the differences were not consistent between time points and similar to previously mentioned parameters have no clinical meaning due to small size of the effect.

Simple regression analysis between the laboratory parameters and clinical data at all investigated time points showed no clinically relevant statistics. (Tables 4–6).

**Table 4:** Results of simple regression analyses between laboratory parameters and clinical data before simulation

Parameters	Cortisol		Testosterone		Alpha-amylase		sIgA	
	*r*	*p*	*r*	*p*	*r*	*p*	*r*	*p*
SBP	−0.227	0.122	−0.038	0.797	0.260	0.075	0.114	0.443
DBP	0.017	0.911	0.219	0.134	0.143	0.333	0.023	0.874
MBP	−0.040	0.788	0.114	0.439	0.199	0.176	0.068	0.647
HR	−0.124	0.401	0.038	0.799	0.036	0.808	−0.033	0.826
SpO_2_	0.057	0.700	−0.236	0.106	−0.281	0.053	−0.100	0.497
Age	−0.134	0.363	0.227	0.121	0.062	0.676	0.051	0.731
Previous simulations [*n*]	−0.264	0.070	−0.122	0.411	−0.028	0.848	0.115	0.438

*sIgA* secretory immunoglobulin A, *TP* total protein, *SBP* systolic blood pressure, *DBP* diastolic blood pressure, *MBP* mean blood pressure, *HR* heart rate, *SpO*_*2*_ blood oxygen saturation

**Table 5:** Results of simple regression analyses between laboratory parameters and clinical data immediately after simulation

Parameters	Cortisol		Testosterone		Alpha-amylase		sIgA	
	*r*	*p*	*r*	*p*	*r*	*p*	*r*	*p*
SBP	−0.101	0.494	−0.158	0.283	0.059	0.693	–0.011	0.939
DBP	−0.146	0.323	0.145	0.326	0.157	0.287	0.194	0.187
MBP	−0.079	0.596	0.159	0.280	0.191	0.194	0.060	0.686
HR	−0.031	0.836	0.208	0.155	0.050	0.738	0.159	0.279
SpO_2_	0.088	0.553	−0.060	0.685	−0.113	0.446	0.040	0.787
Age	−0.186	0.207	0.163	0.267	0.020	0.896	0.092	0.536
Previous simulations [*n*]	−0.214	0.145	−0.086	0.563	−0.095	0.523	0.130	0.378

*sIgA* secretory immunoglobulin A, *TP* total protein, *SBP* systolic blood pressure, *DBP* diastolic blood pressure, *MBP* mean blood pressure, *HR* heart rate, *SpO*_*2*_ blood oxygen saturation

**Table 6:** Results of simple regression analyses between laboratory parameters and clinical data 2 h after simulation

Parameters	Cortisol		Testosterone		Alpha-amylase		sIgA	
	*r*	*p*	*r*	*p*	*r*	*p*	*r*	*p*
SBP	−0.199	0.175	0.259	0.075	0.042	0.776	0.129	0.383
DBP	−0.009	0.954	−0.034	0.820	0.105	0.478	0.125	0.399
MBP	−0.082	0.580	0.043	0.773	0.183	0.214	0.136	0.355
HR	−0.204	0.165	−0.014	0.924	−0.066	0.655	0.052	0.727
SpO_2_	0.342	0.017	−0.519	<0.001	−0.069	0.641	−0.096	0.514
Age	−0.214	0.144	0.195	0.185	0.039	0.795	0.098	0.508
Previous simulations [*n*]	−0.110	0.458	−0.366	0.011	−0.088	0.552	−0.001	0.994

*sIgA* secretory immunoglobulin A, *TP* total protein, *SBP* systolic blood pressure, *DBP* diastolic blood pressure, *MBP* mean blood pressure, *HR* heart rate, *SpO*_*2*_ blood oxygen saturation

## Discussion

In the presented study, we hypothesized that the participants will show an increased stress level after HFS connected with a change in salivary hormone levels. Even though the experience was determined by the students as a source of stress, and this was similar even for subjects with previous experience with HFS, the variation in haemodynamic parameters and difference in concentration of analyzed variables was not statistically significant and it shows rather a trend, that could may be be proven to be significant on a larger group of participants. Cortisol concentration and alpha-amylase activity measured in saliva was higher after the simulation and the concentration of sIgA was reduced at both T1 and T2 time points, which could suggest that the stress level was highest after the simulation, but the size of the effect is small and although this finding is in line with other studies it cannot be stated that in our population this reaction to stress was visible [15]. The hypothalamic-pituitary-adrenal axis and the autonomic nervous system play vital roles in mediating the response to acute stress. Glucocorticoids like adrenocorticotropic hormone and cortisol are activated by the former and catecholamines (adrenaline and noradrenaline) by the latter. These hormones can be measured in saliva, blood, plasma, or urine. It is worth noting that salivary cortisol is a useful biomarker in stress research, together with salivary alpha-amylase, testosterone, and sIgA.

It is also well established in literature that medical students have a higher baseline level of stress and can be exposed to different kinds and levels of stress related to high academic expectations and a rich curriculum. It is of great interest for academic teachers, especially in medical faculties, that too much stress can be associated with adverse effects on cognitive performance. The relationship between stress intensity and the quality of memorizing information follows an inverted U-shaped curve (Yerkes–Dodson law) that defines an ideal performance zone and the zones where performance declines owing to either boredom (when stress is too weak) or distress (when stress is too high) [16]. HFS seems to be an ideal way of teaching medical subjects, eliminating the potential of harming real patients. It appears, however, that its role may be overrated, at least in some areas, as the intensity of stress experienced during HFS can be as high as to lead to a posttraumatic stress disorder [17].

In addition, it was suggested that strong emotional involvement could have a negative effect on the performance of professionals in simulated crisis situations. This negative impact on the ability to perform technical procedures was highlighted with cricothyrotomy or laparoscopy in a study by Evain et al. [18]. In turn, a combination of intense emotional events could improve the mechanisms of memory consolidation. Thus, simulation learning and the resulting emotional involvement can contribute to the retention and consolidation of knowledge and, moreover, the acquisition of appropriate behaviors during adverse events [19]. Boet et al. [20] demonstrated that complex procedural tasks acquired during HFS were retained for at least 1 year after the learning session. However, it is important to add that the retention of skills is incomplete if simulation sessions are associated with high levels of stress [21].

In our study there was a difference in haemodynamic parameters before and after simulation. SBP, MBP, and HR were statistically significantly higher at the T1(immediately after the HFS) than at the T0 and T2 time points (*p* < 0.05), which is a known acute reaction to stress, also reported by numerous authors already cited in this manuscript [9], however as with hormone levels the differences are not strongly pronounced. In our study subject males had significantly higher SBP and lower SpO_2_ values at each time point. This could suggest a different cardiovascular reaction to stress or different coping mechanisms in male medical students but this assumption has to be checked in a specifically designed clinical investigation. Moreover, as all the measurements did not exceed normal values, this difference remains clinically negligible.

Another aspect that should be discussed is an impact of a given simulation scenario on the level of stress in the participants. For the purpose of the study, we aimed to prepare a scenario that was supposed to be extremely stressful. It also involved a patient’s death, which is an issue quite extensively studied in the professional literature. Some investigators, e.g., Leighton [22], proposed several concerns, including students’ feeling guilty over the patient’s death, negative attitudes toward simulation, feelings of inadequacy, and the fact that simulations may bring out buried feelings from the learners’ past. In a survey by Nickerson and Pollard [23], increased emotional distress was reported among participants during a simulation in which an unexpected patient’s death occurred. Also, a study performed by Fraser et al. illustrates a weak statistically significant correlation (*p* < 0.04; 95% CI: 0.14–0.95) between experiencing a simulated patient’s death and worse performance [24].

On the other hand, various investigators, e.g., LeBanc et al. [25], implied that participation in both high- and low-stress simulation scenarios was associated with increased HR compared with baseline and found no significant difference in HR elevation between the high- and low-stress conditions, while cortisol concentrations and subjective stress evaluations were significantly higher in the high-stress scenario. When subjects experienced a simulated patient’s death, peak HR values were significantly higher at the end of the simulation as compared with a group not experiencing death; however, there were no significant differences in salivary stress biomarkers between the groups. Six months later, no significant differences were reported in knowledge or skill testing between the participants who had experienced a simulated patient’s death and those who had experienced a simulated patient’s survival. In the context of clinical learning in this study, the simulated death did not appear to impact learning, the participants’ opinions on simulation, or their performance; neither did it lead to undue stress or feeling of guilt associated with the event after 6 months. These findings prove that dealing with a simulated patient’s death may not necessarily be an additional source of distress and can be applied as a way to prepare participants for future work environment [26].

In our study the assumption was that team leaders would experience more stress than regular participants. This could be explained not only by the leaders’ taking responsibility for the team actions but also by other aspects of the simulation session, such as apprehension about potential evaluation during debriefing, colleague observation, and judgment by peers and instructors [27]. The results of our study did not support this thesis as differences in variables were not significant and not consistent in predefined time points.

We observed a slight difference between male and female participants’ reaction to stress caused by HFS. There was a significant difference in SBP and SpO_2_ between sexes but we did not identify any significant difference in hormone concentrations. This particular finding is unexpected and interesting as it is not in line with other reports as the majority of literature states that the levels of salivary testosterone are usually lower in females than in males. It is possible that female medical students experience more severe stress connected with HFS experience. It had been reported earlier that men and women responded differently to stress [28]. Many studies show that women are characterized by more pronounced trait anxiety than men. This turns out to be valid for medical students as well. The explanation of the difference is poorly understood; a variety of factors, including cultural standards, education, and neurobiology, may play a role.

Identifying students with a higher risk of maladaptive behavior could be crucial for instructors using HFS. The instructors could consider modifying the emotional content of HFS sessions as a function of students’ anxiety. Learners who are more vulnerable could receive more benevolence and reassurance by instructors whereas those who are more naturally relaxed and resilient could be provided with harder stimulation of their cognitive processes [29].

## Conclusions

High-fidelity simulation is a useful tool to bridge the gap between theoretical and practical learning in medical subjects, especially in cases where a mistake could have potentially serious or irreversible consequences. It induces levels of stress comparable with clinical duties and can be stimulating for effective learning but it can also lead to underperformance if the stress is too high. In our study, HFS increased the concentrations of stress hormones, and thus it can be assumed effective as a learning aid even in students of senior years of medical faculty but the results show rather a trend and have to be confirmed on a large group of participants.

Because the difference in hormone levels and haemodynamic parameters were clinically insignificant, we assume that the level of stress connected with HFS, even with a very stressful scenario as presented in our study is not dangerous for the wellbeing of students and that it should not cause any persistent emotional distress. Taking under consideration the results of our study it can be assumed that medical students are more resilient to stressful stimuli and can extensively benefit from this method of gaining knowledge and experience.

## Limitations

Our findings are limited by certain factors. Firstly, the study sample was quite small. Moreover, when measuring hormone concentrations, one should consider such conditions as preexisting endocrine disorders, time of day, and chronic stress, which can all cause variations in the investigated parameters. Also the study would benefit from collecting diurnal basal values of the hormones investigated at a standardized time point, apart from any other special events that could influence the result.

## Supplementary information

Supplementary information

## Data Availability

The data are available at the corresponding author.
