# Supplementary material for: Effect of high-fidelity simulation on alpha-amylase activity and concentrations of secretory immunoglobulin class A, cortisol, and testosterone among medical students
Source: Endocrine. 2021 Apr 5;73(2):431–8. doi: 10.1007/s12020-021-02696-z (PMC8263411; doi:10.1007/s12020-021-02696-z)
Supplement: Supplementary file 1 — Supplementary information [file 12020_2021_2696_MOESM1_ESM.docx]

Table 4. Comparative analysis for team leaders and other members

| Parameter | Leaders | SD | Other members | SD | *p* |
| --- | --- | --- | --- | --- | --- |
| Age [years] | 25 | 1 | 25 | 3 | 0.902 |
| Previous simulations [*n*] | 21 | 16 | 20 | 16 | 0.781 |
| BMI [kg/m^2^] | 21.3 | 2.5 | 22.7 | 3.1 | 0.179 |
| **T0 point** | | | | | |
| SBP [mm Hg] | 126 | 11 | 126 | 14 | 0.994 |
| DBP [mm Hg] | 69 | 9 | 74 | 10 | 0.115 |
| MBP [mm Hg] | 91 | 9 | 95 | 10 | 0.280 |
| HR [bpm] | 82 | 9 | 80 | 14 | 0.699 |
| SpO_2_ [%] | 99 | 1 | 98 | 1 | 0.640 |
| Cortisol [nmol/l] | 74.8 | 15.9 | 108.8 | 33.1 | 0.002 |
| Testosterone [pg/ml] | 98.2 | 29.6 | 114.8 | 32.3 | 0.128 |
| Alpha-amylase [U/ml] | 39.1 | 3.7 | 54.7 | 15.0 | 0.001 |
| sIgA [µg/ml] | 183.7 | 40.3 | 240.6 | 42.8 | 0.000 |
| **T1 point** | | | | | |
| SBP [mm Hg] | 133 | 10 | 132 | 12 | 0.628 |
| DBP [mm Hg] | 74 | 7 | 76 | 10 | 0.394 |
| MBP [mm Hg] | 97 | 8 | 99 | 10 | 0.653 |
| HR [bpm] | 85 | 13 | 90 | 18 | 0.416 |
| SpO_2_ [%] | 98 | 2 | 98 | 1 | 0.674 |
| Cortisol [nmol/l] | 80 | 11 | 110 | 28 | 0.001 |
| Testosterone [pg/ml] | 114.8 | 22.8 | 129.8 | 24.7 | 0.074 |
| Alpha-amylase [U/ml] | 46.0 | 7.4 | 58.8 | 15.3 | 0.009 |
| sIgA [µg/ml] | 168.1 | 8.7 | 241.6 | 45.4 | 0.000 |
| **T2 point** | | | | | |
| SBP [mm Hg] | 122 | 9 | 123 | 12 | 0.844 |
| DBP [mm Hg] | 69 | 8 | 72 | 10 | 0.262 |
| MBP [mm Hg] | 89 | 8 | 92 | 9 | 0.313 |
| HR [bpm] | 76 | 14 | 76 | 12 | 0.976 |
| SpO_2_ [%] | 98 | 1 | 98 | 1 | 0.446 |
| Cortisol [nmol/l] | 79.2 | 14.9 | 113.6 | 30.9 | 0.001 |
| Testosterone [pg/ml] | 128.7 | 21.9 | 143.9 | 23.2 | 0.054 |
| Alpha-amylase [U/ml] | 41.9 | 11.0 | 54.2 | 15.6 | 0.017 |
| sIgA [µg/ml] | 166.9 | 23.7 | 233.2 | 41.7 | 0.000 |

Data presented as mean ± SD. BMI – body mass index, SBP – systolic blood pressure, DBP – diastolic blood pressure, MBP – mean blood pressure, HR – heart rate, SpO_2_ – blood oxygen saturation, sIgA – secretory immunoglobulin A.
